# The impact of subgingival irrigation using ketorolac and chlorhexidine in patients with chronic periodontitis: A randomized, double-blind, controlled, clinical trial

**DOI:** 10.34172/japid.025.2316

**Published:** 2025-05-20

**Authors:** Amirhossein Farahmand, Ali Ghanbarzadeh, Zahra Salmani, Maryam Zohary, Marzieh Ghanbarzadeh

**Affiliations:** ^1^Department of Periodontics, School of Dentistry, Borujerd Medical Sciences, Islamic Azad University, Lorestan, Iran; ^2^Private Practice, Tehran, Iran; ^3^Department of Periodontics, Dental School, Alborz University of Medical Sciences, Karaj, Iran; ^4^Department of Periodontics, Dental School, Guilan University of Medical Sciences, Rasht, Iran

**Keywords:** Anti-inflammatory medicines, Ketorolac, Periodontal diseases, Subgingival irrigation

## Abstract

**Background.:**

Ketorolac is classified as a non-steroidal anti-inflammatory drug. It functions by inhibiting the production of prostaglandins, thereby diminishing the local inflammatory response. This medication has the potential to alleviate postoperative complications, including pain and swelling that may occur following surgical procedures.

**Methods.:**

Fifty patients with mild chronic periodontitis were randomly divided into two blinded groups of 25 patients. One group underwent scaling and root planing (SRP) with 2% ketorolac trometamol (KT) irrigation, and the other group received 0.2% chlorhexidine (CHX). Treatment was performed on the first and second molars in both mandibular quadrants. Various clinical periodontal parameters, such as plaque index (PI), bleeding on probing (BOP), pocket probing depth (PPD), clinical attachment loss (CAL ), and gingival index (GI) were carefully recorded. Patients were scheduled for follow-up visits at 3-month intervals.

**Results.:**

The CHX mouthwash and KT groups did not differ significantly in clinical periodontal parameters at baseline. Clinical outcomes demonstrated, as anticipated, statistically significant improvements in the percentages of PI, BOP, GI, PD, and CAL at 60 and 90 days compared to baseline in both groups (*P*<0.05). In contrast to the CHX group, the KT group’s clinical periodontal parameters (PI, BOP, and GI) significantly decreased after the follow-up period.

**Conclusion.:**

KT can be recommended as a complementary treatment for individuals suffering from chronic periodontitis, as it is more effective in reducing PI, GI, and BOP compared with CHX.

## Introduction

 Periodontitis is a common inflammatory condition primarily caused by the accumulation of microbial pathogens subgingivally, which triggers the host’s immune and inflammatory responses.^1^ The host immune system’s anti-inflammatory cytokines and enzymes play a crucial role in regulating the levels of inflammatory mediators within periodontal tissues. Their primary function is to eliminate microbial pathogens while protecting the host’s health.^2,3^ Numerous research investigations have demonstrated that antagonists of IL-1 and TNF-α impede the progression of inflammatory cells infiltrating the alveolar bone crest. The involvement of osteoclasts and the management of periodontal lesions may lead to the reduction of soluble cytokine antagonists before their peak efficacy, potentially necessitating the additional application of active agents to address periodontal defects.^4^ The variability observed in the host’s response is influenced by environmental and risk factors that can accentuate the host’s inflammatory response. This alteration in the inflammatory process, particularly concerning the host’s response, has led to advancements in host modulator treatments. These treatments can potentially enhance therapeutic outcomes, decelerate disease progression, facilitate more consistent patient management, and possibly serve as preventive measures against the advancement of periodontal diseases. Prostaglandins play a crucial role as the primary mediators of bone loss associated with periodontitis.^5-7^ Non-steroidal anti-inflammatory drugs (NSAIDs) inhibit the activity of cyclooxygenase isoenzymes, specifically COX-1 and COX-2. Numerous studies have demonstrated the efficacy of NSAIDs, including flurbiprofen, indomethacin, and naproxen, in preventing gingivitis and the progression of periodontitis.^8‒10^ The localized administration of NSAIDs to periodontal tissues may provide additional advantages for patients while simultaneously reducing the likelihood of adverse effects. Various topical agents, including flurbiprofen,^11^ ibuprofen,^12^ aspirin,^13^ piroxicam,^14^ tenoxicam,^15^ ketoprofen,^16^ and ketorolac,^17,18^ have demonstrated efficacy in the modulation of inflammatory periodontal conditions. Also, it was similarly reported that mean PGE2 levels were elevated in the placebo group compared to the ketorolac group when patients used 0.1% ketorolac mouth rinses, with a gradual increase observed over the 12 hours in both groups.^19^ This study aimed to explore the effects of sub-gingival irrigation using ketorolac and chlorhexidine (CHX) in individuals suffering from chronic periodontitis.

## Methods

 Fifty patients (15 men and 25 women, 30‒53 years of age) with initial chronic periodontitis were recruited for this splint-mouth double-blind, randomized controlled clinical trial from patients referred to the Broujerd Islamic Azad University of Medical Sciences Faculty of Dentistry and a private periodontal office. The examiner was not informed of the patient’s assignment to the KT or CHX groups, and the patients were blinded to the type of treatment they were randomly assigned to receive (KT or CHX). The researcher knew about the interventions that were used. The Broujerd Islamic Azad University of Medical Sciences’ Institutional Ethics Committee approved the study protocol, which was carried out following the 2013 revision of the 1975 Helsinki Declaration. The 2010 CONSORT guidelines were followed when reporting the study’s findings. NCT03836781 is the study’s official registration number on clinicaltrials.gov. After receiving ethical approval, all the participants were fully informed about the study both orally and in writing, and their informed consent was acquired.

###  Inclusion criteria

1. Age: 30‒53 years
2. Mild-Moderate periodontitis
3. Patients with at least 20 natural teeth
4. Systemically healthy status
5. Localized chronic periodontitis (stage I to II)—defined as having at least 30% of sites with probing depth ≤ 5 mm, clinical attachment loss (CAL) ≤ 1‒3 mm, bleeding on probing (BOP); radiographic bone loss: extending to the middle (15‒20%).^20^

###  Exclusion criteria

1. Smoking
2. Pregnancy and nursing
3. Using antibiotics locally or systemically for the preceding three months
4. Long-term use of non-steroidal anti-inflammatory drugs
5. Any periodontal treatment during the previous year
6. Systemic disorders (e.g., diabetes mellitus, cancer, immune system disorders, bone metabolic disorders, diseases affecting healing potential)
7. Radiotherapy and immunosuppressive therapies.
8. History of hypersensitivity to ketorolac and CHX
9. Not willing or refusing to sign an informed consent form

###  Patient grouping

 A double-blind, randomized, split-mouth clinical experiment was designed with two parallel groups. Sixty patients were recruited based on eligibility criteria (stages I to II of chronic periodontitis), and those who agreed to participate were randomly assigned to either the ketorolac or CHX groups after being enrolled by the investigators. A basic randomization technique was carried out using the randomiation tool software. Furthermore, mandibular quadrants were assigned randomly, considering the time of patients’ visits and the location of the first and second mandibular molars on both sides of the jaw (left or right quadrant). Thus, each side of the mandibular quadrant was randomly selected, and a different drug was chosen for each side. The investigators were not involved in or aware of the randomization mechanism used to analyze the study outcomes.

###  Intervention

 After collecting baseline data, the periodontist conducted an initial scaling and root planing (SRP) with polishing. This involved root planing and supragingival and subgingival scaling of the entire mouth with curettes and an ultrasonic scaler. Patients also received oral hygiene instructions, such as tooth brushing (Bass technique) and interdental hygiene (dental flossing). Before the trial began, periodontal parameters were also examined for each form of treatment. Following mechanical debridement in both groups, a vial of ketorolac trometamol (KT) (30 mg/mL) (Exir Pharmaceutical Company, Borujerd, Lorestan, Iran) and 0.2% CHX (Ghol Darou, Tehran, Iran) was injected into the pockets surrounding the mandibular teeth on one side in the CHX and KT groups. Both medication bottles were sealed to prevent consumers from seeing the contents to blind the study. Subgingival irrigation was performed using a sterilized insulin syringe and a blunt needle, with drugs administered in 2-mL doses every two weeks. Two mL of each test solution was drawn into the syringe after making a 1-mm mark with a needle tip. The teeth were initially isolated using a cotton roll to ensure the treatment’s validity. To ensure that the rinse fluid was equally dispersed throughout the periodontal pocket, the needle was carefully inserted 1 mm deep. Meanwhile, the first phase filled both groups’ pockets with the rinse solution for two minutes.

 Following treatment, patients were scheduled for follow-up appointments: two weeks, one month, two months, and three months. For three months, this procedure was repeated every fifteen days. The patients did not receive prescriptions for antibiotics or anti-inflammatory medications after their treatment was over. They received detailed instructions for a week, including using any interdental aids, brushing close to the treated regions, and avoiding biting on hard or sticky food. All clinical parameters were assessed once more in the same location for both groups one and three months following the intervention.

###  Periodontal measurement/recording 

 The evaluation involved recording various periodontal clinical parameters such as plaque index (PI), BOP, PD, CAL, and gingival index (GI) at different time intervals: baseline (before mechanical debridement) and throughout the study at 1 month and 3 months. Subsequently, the two periodontists evaluated the subgingival cleaning and its effects on clinical periodontal parameters. In addition, two calibrated blinded examiners used periodontal probes (Williams Probe, Hu-Friedy, USA). They characterized pocket depth as the distance from the gingival margin to the bottom of the pocket and defined CAL as the distance from the cementoenamel junction (CEJ) to the bottom of the pocket. The Silness and Loe PI, which measures plaque accumulation, was used for the evaluation.^21,22^ Additionally, the presence of BOP was measured using a scoring system developed by Carter and Barnes, with a score of 0 indicating no bleeding after probing and a score of 1 indicating bleeding at a single, separate site after probing.^23^ The GI of Loe and Silness (1963) was used to assess the degree of gingival inflammation.^24^

###  Primary and secondary outcomes

 The primary outcomes of the current study were the GI and BOP. The secondary outcomes were PI, CAL, and PD.

###  Sample size

 The sample size was established based on prior research, considering the restrictions, the 1.65 mm pocket depth difference, and an average standard deviation of 1.40. As recommended by Preshaw et al,^19^ the power (β) was set at 0.2 and the significance threshold (α) at 0.05. Using the Mini Tab software, it was determined that a minimum sample size of 50 was needed for both groups; because of a 15% possibility of sample dropout and three follow-up stages, the ultimate sample size was increased to 50, with 25 participants in each group.

###  Statistical analysis

 Statistical analysis was conducted using SPSS 20 to thoroughly examine the data through appropriate statistical methods. The comparison of medicine groups was performed within the same group using the t-test for both baseline measurements and significance after three months, while intergroup comparisons at baseline were assessed using a chi-squared test, maintaining a significance threshold of 0.05. To evaluate the differences in PI following the three-month treatment period, a one-way analysis of covariance (ANCOVA) was employed, with treatment as the independent variable and baseline clinical parameters as covariates, with a significant level of *P* < 0.05. The Mann-Whitney U test was applied to compare the bleeding index between the two groups, while the changes in probing pocket depth and CAL were analyzed using the repeated-measures ANOVA, taking into account the study’s subject factors.

## Results

###  Descriptive results

**Figure 1:**
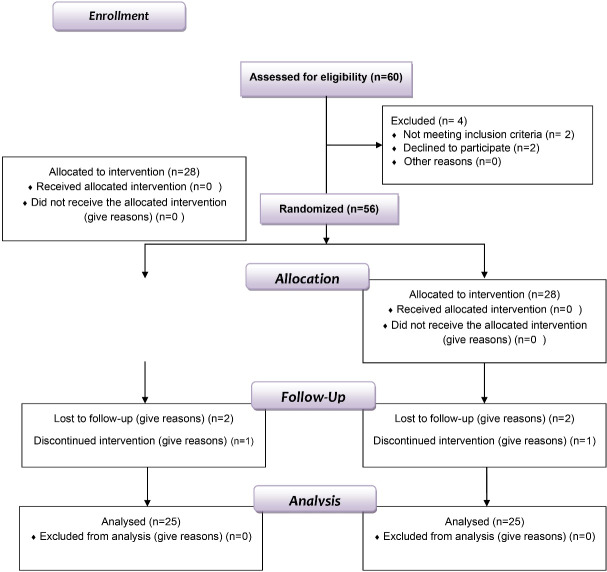
Flow chart of the study design

 Out of 56 participants, 50 (one site/patient) completed the trial successfully. Regretfully, six people could not attend the follow-up sessions—three from the KT group and three from the CHX group (Figure 1). Therefore, after completing the 3-month follow-up, only 50 patients (20 men and 30 women) aged 30‒52 were included in the data analysis (Table 1). Like other NSAIDs, ketorolac did not cause allergic or hypersensitivity reactions when administered topically during the study. It was well tolerated by patients without side effects. During clinical examination and continued study, no patient complained of any discomfort, and no symptoms were observed in the examined area.

###  Clinical parameters

####  Inter-group results

Tables 2 to 6 demonstrate the distributions of the clinical parameters (PI, GI, BOP, PD, and CAL) during the baseline and follow-up visits. Each group’s examined periodontal parameters were less than they had been on the first day. However, the BOP and gingival indices for the KT and CHX groups did not differ significantly (*P* > 0.05) after a month, according to the independent t-test. However, after two and three months, it considerably decreased (*P* < 0.0001) in the KT group; also, the PI gradually declined, although this change was evident in the KT group at two and three months higher than others (*P* > 0.05). However, after three months, both before and after the intervention period, there was no noticeable change in PD between the two groups (*P* < 0.05).

####  Intra-group results

 All patients’ initial periodontal clinical parameters were recorded and evaluated at the beginning and during the three months. Tables 2 to 6 provide intragroup assessments of all periodontal clinical indicators at baseline and one, two, and three months of follow-up. Intragroup comparisons of PI showed that both groups had significant differences in PI at the 3-month follow-up (*P* < 0.05), although there were no statistically significant variations in clinical parameters at baseline. Intergroup comparisons revealed that the KT group’s PI was significantly different (*P* < 0.05) at the 3-month follow-up (Table 4). Furthermore, when comparing the two groups, the KT group showed significant differences in GI and BOP values at the 3-month follow-up (*P* < 0.05) (Tables 2 and 3). Furthermore, a significant reduction in BOP was observed during subgingival cleaning in KT (87%) and CHX (72%); as a result, it was demonstrated that the ketorolac group experienced a 15% reduction in the bleeding index (Table 2). Regarding the GI (Table 3), KT showed a decrease of 81%, and CHX showed a decline of 70%. Consequently, it was discovered that the ketorolac group exhibited a 9% reduction in the GI compared to the CHX group, while the PI also decreased by 73.5% in the KT group and 70% in the CHX group (Table 4). Furthermore, the PPD and CAL decreased by 80% in both groups (KT and CHX groups) and 82%, 69%, and 70% in the CHX group, respectively. Although there were no significant differences between the two groups regarding PPD and CAL at 3-month follow-up, these paired results indicated a slightly positive impact of KT on clinical parameters compared to CHX.

**Table 1:** Demographic data of the patients

**Demographic data**	**No.**	**Percent**	
Gender			
Male	15	37.5	
Female	25	62.5	
	**No.**	**Mean±SD**	**Range**
Age groups (years)			
30‒39	22	32.3 ± 2.3	30-35
40‒49	14	41.5 ± 1.5	40-43
50‒59	4	52.6 ± 2.6	50-55

**Table 2:** Mean bleeding on probing (BOP) at baseline and 1‒3 months after probing in the KT and CHX groups

**BOP**	**Baseline**	**30 days**	**60 days**	**Final**
Chlorhexidine	81.7 ± 22.1	51.20 ± 18.70	30.60 ± 0.56	22.43 ± 0.18
Ketorolac	82.5 ± 12.3	45.67 ± 12.80	23.46 ± 0.11	11.70 ± 0.14
*P* value	0.8883	0.2733	0.0001*	0.0001*

* These changes are considered statistically significant intervals between the two groups after the three-month study (*P* ≤ 0.05).

**Table 3:** Mean gingival index (GI) for the KT and CHX groups at baseline and after 1–3 months

**GI**	**Baseline**	**30 days**	**60 days**	**Final**
Chlorhexidine	1.65 ± 0.16	0.97 ± 0.37	0.56 ± 0.36	0.48 ± 0.26
Ketorolac	1.66 ± 0.18	0.87 ± 0.39	0.41 ± 0.39	0.31 ± 0.32
*P* value	0.7697	0.1915	0.0484*	0.0447*

* This difference is considered a statistically significant interval between both groups after the 90-day evaluation (*P* ≤ 0.05).

**Table 4:** Mean plaque index (PI) in KT and CHX groups at baseline and 1–3 months

**Groups PI**	**Baseline**	**30 days**	**60 days**	**Final**
Chlorhexidine	0.89 ± 0.21	0.54 ± 0.26	0.38 ± 0.09	0.26 ± 0.10
Ketorolac	0.88 ± 0.22	0.50 ± 0.22	0.30 ± 0.06	0.17 ± 0.01
*P* value	0.8839	0.6025	0.0001*	0.0101*

* This difference is considered a statistically significant interval between the two groups at the study’s endpoints (*P* ≤ 0.05).

**Table 5:** Periodontal pocket depth (PPD) variables of the subjects at baseline to 3 months

**PPD**	**Baseline**	**30 days**	**60 days**	**Final**
Chlorhexidine	5.15 ± 0.48	4.45 ± 0.18	4.35 ± 0.14	4.13 ± 0.15
Ketorolac	5.18 ± 0.47	4.50 ± 0.15	4.38 ± 0.12	4.15 ± 0.17
*P* value	0.8243	0.1345	0.2528	0.6611*

* The difference between the two groups was not statistically significant at baseline and the 12 weeks (*P* ≤ 0.05)

**Table 6:** Clinical Attachment loss (CAL) variables of the subjects at baseline to 3 months

**CAL**	**Baseline**	**30 days**	**60 days**	**Final**
Chlorhexidine	5.56 ± 0. 34	5.15 ± 1.80	4.34 ± 0.13	4.20 ± 0.11
Ketorolac	5.60 ± 0.48	5.20 ± 1.60	4.38 ± 0.15	4.24 ± 0.14
P value	0.7627	0.9084	0.1574	0.1154*

* These changes between the two groups were not statistically significant at baseline and after 12 weeks (*P* ≤ 0.05).

## Discussion

 Anti-inflammatory drugs have been used as adjuncts to non-surgical periodontal treatment. However, the efficacy of these agents in periodontal treatment remains controversial. Using a clinical trial, this study investigated the anti-inflammatory effect of KT as an adjunct to non-surgical periodontal treatment. This study showed a statistically significant difference in GI reduction between CHX and KT as an adjunct to non-surgical periodontal treatment at the 2- and 3-month follow-up periods. A significant difference was also found in the BI reduction between these two groups in the 2nd and 3rd months. Similarly, Jeffcoat et al^18^ showed that using 0.1% KT as a mouthwash exerted beneficial therapeutic effects, including preventing alveolar bone loss. The primary outcome measure was the BOP index; furthermore, all sites treated with non-surgical periodontal therapy showed improvements after 3 months.

 Farahmand et al^16,25^ showed that using ibuprofen gel as a subgingival irrigation solution significantly reduced BOP compared to the placebo group. Also, Paquette et al^26^ and Srinivas et al^27^ described a reduction in inflammatory components after applying similar ketoprofen. Furthermore, Heasman et al^11^ found that clinical gingival bleeding indexes were significantly reduced in periodontitis patients treated with NSAIDs, further supporting this understanding of the BOP results. Also, Howell et al^10^ and Heasman et al^8,11,28^ reported that the use of anti-inflammatory agents reduced GI in the test group compared to the control group. Furthermore, Feldman et al^29^ found that statistically significant differences were only observed in the GI when only patients treated with indomethacin were evaluated. Thus, based on numerous studies, ketorolac is the leading non-steroidal anti-inflammatory drug proposed to improve many parameters of periodontitis. This drug is used for severe and moderate pain in periodontitis postoperatively.^18,19,30,31^ It has been demonstrated that in periodontitis, increased IL-1 production leads to the activation of prostaglandin E2 (PGE2), including the induction of matrix metalloproteinase production. This mechanism causes tissue inflammation and is associated with bone resorption.^32^ Yang et al^33^ used KT gel and ketorolac trometamol gel containing genipin (KTG gel) to study their therapeutic effects on periodontitis. KTG gel is believed to be effective against gingival pocket gingivitis due to its increased anti-inflammatory effect and cross-linking between genipin and biological tissues. However, recent studies suggest that using NSAIDs in conjunction with non-surgical periodontal treatment may provide further improvements in periodontal disease by modulating the host immune‒inflammatory response.^34-36^ On the other hand, the use of ketorolac has had a positive effect on the treatment of periodontitis and may be beneficial^36^ Rosin et al^37^ noted no statistically significant difference in GI between placebo and dexibuprofen. However, the reduction in Quigley & Hein plaque index (QHI) was significantly greater with dexibuprofen compared with placebo; additionally, in this study, a 1.5% dexibuprofen mouthwash did not affect gingivitis, but an antiplaque effect was demonstrated. Moreover, Sekino et al^38^ found that patients accumulated large amounts of plaque and developed significant signs of gingival inflammation while rinsing with saline. When they rinsed with CHX, a small amount of plaque formed and only a few sites reached a GI score of ≥ 2. After 2 weeks of ibuprofen treatment, participants had a significant reduction in the number of sites with a GI score of ≥ 2, but the same amount of plaque had formed as during the negative control period. On the other hand, CHX is an antiseptic. CHX is useful for its broad-spectrum antibacterial activity and is substantial, safe, and non-toxic. It has also been used to treat periodontitis over the past 40 years. However, subgingival irrigation with CHX has not been effective in treating periodontitis due to the lack of an effective concentration and the unique nature of the anatomical structure of the gingival pocket.^39^ In any case, when CHX is used as a mouthwash, side effects such as changes in the color of the teeth, teeth and mucous membranes, dryness and pain of the mucous membranes, changes in taste, and increased plaque formation on the gums may be observed.^40^ Meanwhile, previous studies such as that by Soh et al^41^ demonstrated that subgingival irrigation with CHX effectively reduced inflammation associated with periodontitis and facilitated plaque control. Furthermore, Asari et al^42^ reported that subgingival irrigation with CHX significantly improved clinical parameters in treating periodontitis. Southard et al^43^ also reported that a combined approach of SRP and subgingival irrigation with CHX four times per week resulted in increased attachment again and a longer-lasting reduction in *P. gingivalis* compared with SRP or subgingival irrigation alone. This decrease in PI could be attributed to the anti-inflammatory characteristics of ketorolac. Furthermore, a study by Cosyn et al^44^ suggest that solutions and gels may not be an adequate substitute when SRP is insufficient but suggest that complementary chemotherapy with subgingival CHX irrigation may be beneficial. Research by Gebaraa et al^45^ indicated that subgingival irrigation with propolis extract as an adjunct to periodontal therapy was more effective than conventional treatments, based on both clinical and microbiological criteria. In contrast, Braatz et al^46^ reported that daily use of CHX irrigation in deep periodontal pockets did not enhance the outcomes of non-surgical periodontal treatment. Also, MacAlpine et al^47^ stated that bi-weekly deep pocket irrigation with CHX, tetracycline, or saline does not appear to enhance the efficacy of non-surgical periodontal treatments. However, 0.12–0.2% CHX has traditionally been used as an adjunct to SRP to control various periodontal inflammatory diseases. Moreover, a systematic review examining the impact of subgingival irrigation with CHX found no additional benefits over mechanical debridement.^48^ Two studies^49,50^ have found that 0.2% CHX exhibits little or no antibacterial activity against various enteric gram-negative rods and oral biofilm microorganisms. A similar study showed that CHX impairs fibroblast morphology.^51^ Furthermore, Zhao et al^52^ and Poppolo^51,53^reported that as a non-surgical periodontal treatment for periodontitis, additional subgingival application of CHX gel at concentrations of 0.5% to 2.0% yielded a slight advantage in periodontal pocket depths of ≥ 4 before probing. Yuan et al^54^ described that the treatment outcomes of chronic periodontitis could be improved by treating the root surface with simultaneous ultrasonic scaling and CHX irrigation. The adjunctive use of 0.12% CHX with a newly designed ultrasonic scaler tip in treating moderate-to-severe chronic periodontitis demonstrated significant clinical benefits and decreased inflammatory mediators compared to scaling and root planing plus placebo. Moreover, Lecic et al^55^ demonstrated significant improvements in the BI and PPD in the CHX chip with the SRP group compared to the SRP-only group at the three-month follow-up. These findings support the use of combination therapy involving a CHX chip as an adjunct to SRP, as it yields superior results in BI and PPD in managing chronic periodontitis compared to SRP alone. Annisa et al^56^ also reported that CHX chips showed superior efficacy on the GI compared to other antimicrobials over three months. Other antimicrobials demonstrated more efficacy than CHX chips in reducing probing depth after one and three months and surpassed CHX gels in lowering PI after one month. Susanto et al^57^ reviewed CHX for subgingival irrigation, noting that incorporating CHX into SRP offers extra clinical advantages over SRP alone in treating chronic periodontitis. However, by understanding the properties and limitations of the CHX molecule, the dental profession can ensure that the agent’s efficacy is maximized while the side effects are minimized, allowing CHX to remain the gold standard against which other antiplaque agents are measured.^58^ Therefore, using antibiotics and anti-inflammatory drugs locally is more beneficial than using these agents systemically. However, local agents used in subgingival irrigation may affect periodontal pathogens deep in periodontal pockets, tooth furcation, and other inaccessible areas. Furthermore, most of the agents available for subgingival irrigation do not have long-term efficacy. Also, subgingival irrigation as an adjunct to traditional periodontitis treatment has produced mixed results. According to Allison et al,^59^the NSAID used in this study was KT. It proved to be a more potent bone resorption inhibitor than other NSAIDs such as flurbiprofen, naproxen, piroxicam, and ibuprofen. Kelm et al^31^ used KT locally as an active ingredient in mouthwash and toothpaste. Their results also showed that the concentration of KT in GCF was high enough to inhibit PGE2 production. However, the above results suggest that the reduction in BOP and GI may be justified by the anti-inflammatory properties of this drug. Although regular periodontitis treatment is the most effective method, we believe that using local agents can help reduce the clinical symptoms of periodontitis. Therefore, we recommend using local agents, as this change in the patient’s health status acts as a complement to periodontitis treatment, affecting the quality and quantity of plaque and altering the inflammatory process in the periodontal tissues.

## Conclusion

 CHX is a highly efficient antibacterial agent in the field of health. In dentistry, its versatility as a chemotherapeutic agent is unparalleled when mechanical prophylaxis is not possible; the available CHX concentration is also recommended to vary between 0.12% and 0.2%. CHX mouthwash is preferred over gels and dentifrices because it inhibits plaque more effectively and has no negative side effects. CHX mouthwash is the most often used and is regarded as a gold-standard chemical agent. However, using KT and other therapeutic agents in subgingival irrigation may open up new horizons for the non-surgical treatment of chronic periodontitis. The outcomes of this study suggest that KT may be a viable alternative to CHX, especially since it demonstrated excellent efficacy in reducing BOP and the GI of chronic periodontitis.

## Competing Interests

 The authors confirm that they do not have any competing interests regarding the authorship and/or publication of this study.

## Data Availability Statement

 Interested individuals can obtain the necessary raw or processed data to replicate these findings by contacting the corresponding author after the publication.

## Ethical Approval

 The research conducted in this study was approved by the Human Research Ethics Committee of Borujerd University of Medical Science, Lorestan, Iran, under the code 8769.
